# Selective leaf surface defenses: trichomes trap herbivorous leafminers but spare parasitoid wasps

**DOI:** 10.1002/ps.70523

**Published:** 2026-01-15

**Authors:** Yuta Ohata, Yuhko Sawada, Yuki Ishihara, Yohsuke Tagami

**Affiliations:** ^1^ Gifu University, United Graduate School of Agricultural Science Gifu Japan; ^2^ Faculty of Agriculture Shizuoka University Shizuoka Japan; ^3^ Fisheries Resources Institute Japan Fisheries Research and Education Agency Yokohama Japan

**Keywords:** morphological adaptation, non‐glandular trichomes, plant trichome defense, selective pest control

## Abstract

**BACKGROUND:**

Leafminers [e.g., *Liriomyza trifolii* (Burgess), *L. sativae* (Blanchard), and *Chromatomyia horticola* (Goureau)] are globally significant agricultural pests, while their parasitoid wasps [e.g., *Hemiptarsenus varicornis* (Girault) and *Neochrysocharis formosa* (Westhood)] are key agents in biological control programs. Although trichomes are known to act as physical defenses against herbivores, their potential for selective entrapment—targeting pests while sparing natural enemies—has remained underexplored. This study aimed to evaluate whether kidney bean (*Phaseolus vulgaris* L.) leaf trichomes exert species‐specific physical effects on leafminers and their parasitoids.

**RESULTS:**

Laboratory assays showed that 30–40% of adult leafminers became entangled and died on the abaxial surface of primary leaves. In contrast, only 1.6–3.3% of parasitoid wasps were similarly affected. Scanning electron microscopy revealed that hook‐shaped non‐glandular trichomes effectively trapped leafminer legs, ovipositors, and mouthparts, regardless of sex or age. In contrast, parasitoids avoided entrapment, consistent with their markedly thinner legs and smaller body size. Removal of trichomes drastically reduced leafminer attachment but did not reduce and increase parasitoid parasitism efficiency.

**CONCLUSION:**

Kidney bean plants exhibit a selective physical trapping mechanism that disproportionately affects herbivorous pests while sparing their parasitoid natural enemies. This species‐specific interaction, governed by trichome morphology and insect body structure, provides an example of a plant defense trait that is compatible with biological control. These findings highlight the potential for leveraging natural plant defenses to enhance the selectivity and ecological compatibility of integrated pest management programs. © 2026 The Author(s). *Pest Management Science* published by John Wiley & Sons Ltd on behalf of Society of Chemical Industry.

## Graphical abstract

Hooked trichomes on kidney bean leaves selectively entrap leafminer flies but rarely affect parasitoid wasps. This morphological barrier enables pest suppression with minimal impact on beneficial insects, offering insights for breeding pest‐resistant cultivars compatible with biological control.

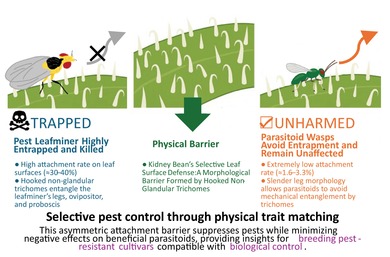

## 1 INTRODUCTION

Plants possess a wide array of surface structures that function in defense, water retention, UV protection, and other ecological roles.^1^ Among these, trichomes—hair‐like epidermal outgrowths—are known to serve as physical barriers that influence insect attachment, movement, and survival.^2^, ^3^ Trichomes vary widely in shape, size, and density across plant species and tissues, and have been shown to deter herbivorous insects through mechanical entrapment or behavioral interference. The spatial distribution of trichomes, particularly on the adaxial (upper) and abaxial (lower) leaf surfaces, may strongly affect insect surface preference and behavior.^4^

Leafminers (Diptera: Agromyzidae) are phytophagous insects that feed within leaf tissues and cause significant damage to agricultural and ornamental crops worldwide.^5^ In Japan, three economically important species—*Liriomyza trifolii* (Burgess), *L. sativae* (Blanchard), and *Chromatomyia horticola*(Goureau)—frequently infest kidney bean plants (*Phaseolus vulgaris* L.), among other hosts.^6^, ^7^ Female leafminers insert their eggs into leaf tissues using their ovipositors and subsequently feed on plant fluids via punctures. *L. trifolii* adults are frequently trapped by leaf surface trichomes during landing, feeding, or oviposition, particularly on the abaxial leaf surface, where trichomes are denser.^8^

Biological control of leafminers relies on a diverse assemblage of parasitic wasps from various Hymenopteran families, including Eulophidae, Braconidae, Pteromalidae, and Figitidae.^9^, ^10^, ^11^, ^12^, ^13^ In Japan, *Neochrysocharis formosa* (Westwood), *Hemiptarsenus varicornis* (Girault), and *Chrysocharis pentheus* (Walker) are important native parasitoids of leafminers.^14^, ^15^ These parasitoids parasitize the larval stage of multiple leafminer species—including *L. trifolii*, *L. sativae*, and *C. horticola*—by ovipositing through the leaf epidermis.^9^, ^10^, ^16^, ^17^, ^18^ As both leafminers and their parasitoids walk across and interact with the same leaf surfaces during host location and oviposition,^19^, ^20^, ^21^ trichomes may affect both groups, potentially influencing the efficacy of biological control.

Although many studies have documented the defensive role of trichomes against herbivorous insects,^3^, ^22^, ^23^ fewer have examined their effects on beneficial arthropods such as parasitoids.^24^, ^25^ In some systems, dense or glandular trichomes have been shown to reduce parasitoid efficacy by impeding host searching or oviposition.^25^, ^26^ Such findings suggest a potential trade‐off between direct defense against herbivores and indirect defense via natural enemies.^27^ However, few studies have quantitatively compared the effects of trichomes on herbivores and their parasitoids within a shared experimental framework.

In this study, we used the kidney bean plant as a model plant and investigated the role of its hook‐shaped, non‐glandular trichomes in mediating species‐specific entrapment. We focused on three insect groups: leafminers (*L. trifolii*, *L. sativae*, *C. horticola*), their parasitoid wasps (*N. formosa*, *H. varicornis*), and a non‐associated control species, *Drosophila melanogaster* (Meigen). We aimed to (i) quantify trichome density across different leaf regions, (ii) measure attachment and mortality patterns among insect species, (iii) assess the functional relevance of trichomes using removal treatments, and (iv) compare morphological traits related to attachment, such as leg width. Our results shed light on how structural plant traits modulate plant–insect interactions at micro‐scales, with implications for selective pest management and banker plant strategies.^28^

## 2 MATERIALS AND METHODS

### 2.1 Insects and plants

The leafminer *L. trifolii* was collected in 1991, in Hamamatsu, Shizuoka, Japan, *L. sativae* was collected in 2004, Iwata, Shizuoka, Japan, and *C. horticola* was collected in 2005, Iwata, Shizuoka, Japan. These three species were reared into a cage (22 cm width × 35 cm depth × 35 cm height) at 25, 23, and 20 °C, respectively and under 16 h light:8 h dark (16L:8D) photoperiod conditions. The kidney bean was used as the host plant for these species. *L. trifolii* and *L. sativae* primarily perform both feeding and oviposition on the adaxial leaf surface of kidney bean plants. In contrast, *C. horticola* females typically feed on the adaxial surface but oviposit on the abaxial surface of kidney bean plants.

The leafminer parasitoids *H. varicornis* were collected in 2007, in Iwata, Shizuoka, Japan. *N. formosa* used in this study was a thelytokous strain whose *Rickettsia* infection status had been confirmed in a previous study.^29^ This strain was originally collected in 2004, Iwata, Shizuoka, Japan. Although both thelytokous and arrhenotokous strains are known to occur in this species,^30^, ^31^ our experiments exclusively employed the thelytokous strain. Both parasitoid wasps were maintained in the laboratory using *L. trifolii* larvae as hosts under identical rearing conditions (25 °C and 16L:8D).

Kidney bean was used when its primary leaves were sufficiently developed. Primary and true leaves were used in all experiments. The kidney bean was caltivated in a plant pot under conditions of 20 °C and 16L:8D.

### 2.2 Trichome density and insect attachment site

Trichome density was measured on both the adaxial and abaxial surfaces of the primary and true leaves using scanning electron microscopy (SEM; JSM6060, JEOL, Tokyo, Japan). To assess the attachment preference of insects to plant parts, six kidney bean plants were placed inside an acrylic cage (22 cm width × 35 cm depth × 35 cm height). Then, 30 adult *L. trifolii* or *N. formosa* individuals were released into the cage. Parasitoids were supplied with a 10% honey–water solution applied to the walls to improve survival. The number and position of dead individuals on the plants were recorded after all insects had died. Each trial was replicated 10 times.

### 2.3 Assessment of sex‐ and age‐based differences in attachment

To evaluate sex‐based differences in attachment, 30 adult *L. trifolii* individuals of either sex (males or females) were released into a cage per replicate. Each treatment was replicated 10 times. For age‐based analysis, newly emerged adults were collected daily and maintained on kidney bean plants until they reached the target age (1–8 days post‐eclosion). After reaching the designed age, adults were released under identical experimental conditions and attachment positions were recorded 24 h later.

### 2.4 Microscopic examination of leaf surface structure, insect attachment, and hind tibia thickness

Fully developed primary and true leaves of kidney bean plants were excised with scissors and examined on both the adaxial and abaxial surfaces using SEM. Leafminers attached to the leaf surfaces, as well as the legs of both leafminers and parasitoid wasps, were also observed using SEM. The hind tibia thickness of *L. trifolii* and *N. formosa* was measured on 20 dried specimens of each group using a stereoscopic microscope (MZ16; Leica, Wetzlar, Germany).

### 2.5 Comparison of attachment and oviposition in *L. trifolii* with and without trichomes

To evaluate the effects of trichomes on *L. trifolii* attachment and oviposition behavior, trichomes were carefully removed from both sides of the true leaves of kidney bean plants using a razor blade and forceps, taking care to avoid damaging the leaf surfaces. The cotyledons were removed beforehand, and one plant was placed in a cage. Thirty *L. trifolii* adults were released into each cage and after all individuals had died, their attachment positions on the leaves were recorded. As a control, plants were similarly defoliated of cotyledons and brushed gently on the true leaves with a soft paintbrush to simulate physical stimulation without removing trichomes.

In a separate experiment, three plant treatments were compared: (i) plants with only cotyledons remaining, (ii) plants with only intact true leaves, and (iii) plants with trichomes removed from true leaves. One plant per treatment was placed in a separate cage, and thirty *L. trifolii* adults (15 males and 15 females) were released into each. After 48 h, plants were collected and the number of oviposition punctures on each leaf was counted under a stereoscopic microscope. Leaf area was measured using ImageJ software and oviposition density was calculated as the number of punctures per square centimeter. All treatments were replicated 10 times.

### 2.6 Comparison of parasitoid efficiency in *N. formosa* with and without trichomes

To evaluate whether the presence of trichomes affects the parasitism efficiency of *N. formosa*, we compared parasitism rates across three plant treatments: (i) plants with only intact primary leaves remaining, (ii) plants with only intact true leaves remaining, and (iii) plants with trichome‐removed true leaves. Trichomes were removed from true leaves using the same procedure described in section 3.5. Kidney bean plants infested with *L. trifolii* larvae were prepared for each treatment, and the number of larvae on each plant was counted prior to the experiment. One plant was placed in a cage and 10 female *N. formosa* adults were released into each cage. Seven days after the release, the plants were collected and the number of *N. formosa* mummies (pupae) on each leaf was counted. The experiment was replicated 10 times for each treatment. The parasitism rate was calculated as the number of mummies divided by the initial number of host larvae per replicate.

### 2.7 Comparison of attachment rates among insect species

Six kidney bean plants were placed in a mesh cage (22 cm width × 35 cm depth × 35 cm height). Approximately 30 adults of each insect group—leafminers (*L. trifolii*, *L. sativae*, and *C. horticola*), parasitoid wasps (*N. formosa* and *H. varicornis*), or an ecologically unrelated species (*D. melanogaster*)—were released into the cage. All individuals were less than 3 days post‐eclosion and maintained under controlled conditions (25 °C, 16L:8D photoperiod). To improve survival, 10% honey–water solution was applied to the inner cage walls for parasitoid wasp and *D*. *melanogaster* treatments. In contrast, leafminers were not provided with honey because they naturally fed on kidney bean leaves inside the cage. After all individuals had died, their attachment positions on the plant were recorded. The recorded data represent their final attachment sites at the time of death.

### 2.8 Statistical analyses

All statistical analyses were conducted in R [v4.3.2^32^]. Normality and homogeneity of variances were assessed using the Shapiro–Wilk and Levene's tests, respectively. Parametric tests were applied when assumptions were met; otherwise, non‐parametric methods or generalized linear models (GLMM) were used. The significance level was set at *α* = 0.05.

Trichome densities were analyzed using a Poisson GLMM with leaf type and surface as fixed effects, and replicate as a random effect. *Post hoc* Tukey's honestly significant difference (HSD) tests were conducted where interactions were significant. Attachment counts in *L. trifolii* were analyzed using negative binomial regression to account for overdispersion in high‐attachment groups (e.g., true leaf abaxial surface, where variance exceeded the mean). Subsequent pairwise comparison**s** were performed with Bonferroni correction. Attachment in parasitoids was qualitatively reported due to complete zero values.

Sex and age effects were tested using *χ* ^2^ tests. Hind tibia thickness was compared among groups using the Kruskal–Wallis test, with Bonferroni‐corrected Dunn's test. For the trichome‐removal assays, Wilcoxon rank‐sum tests were used as the primary method because of the small sample size and underdispersed counts (dispersion statistic <1). Additionally, complementary negative binomial models were applied to ensure analytical consistency with the previous attachment analysis, allowing for the estimation of effects. Oviposition densities and parasitism rates among the three treatments were analyzed by Kruskal–Wallis tests. Species‐level attachment rates were compared using a *post hoc* multiple comparison test (Ryan–Einot–Gabriel–Welsch method^33^).

## 3 RESULTS

### 3.1 Differences in trichome density and insect attachment patterns across leaf surfaces

**Figure 1:**
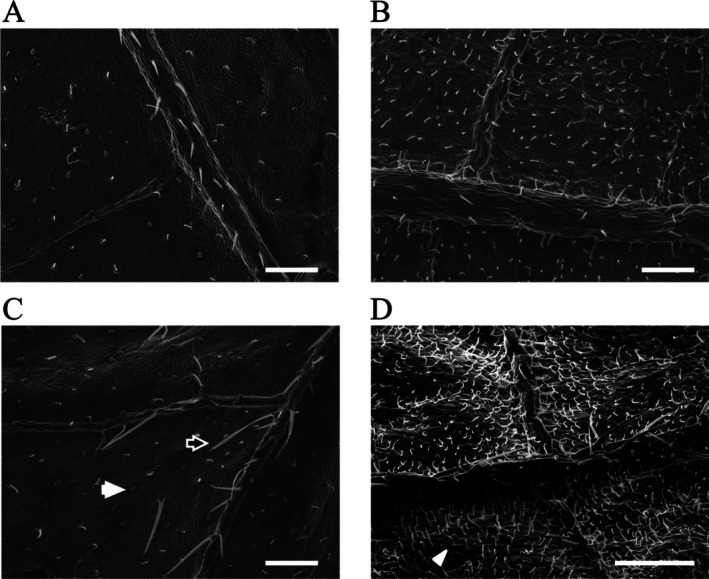
Scanning electron microscopy images of trichomes on different parts of common kidney bean leaves. (A) Adaxial surface of the primary leaf. (B) Abaxial surface of the primary leaf. (C) Adaxial surface of the true leaf. (D) Abaxial surface of the true leaf. Three trichome morphotypes were observed: needle‐shaped (white‐bordered black arrows), glandular (white arrows), and hook‐shaped (white arrowheads). Scale bars: 500 μm.

**Figure 2:**
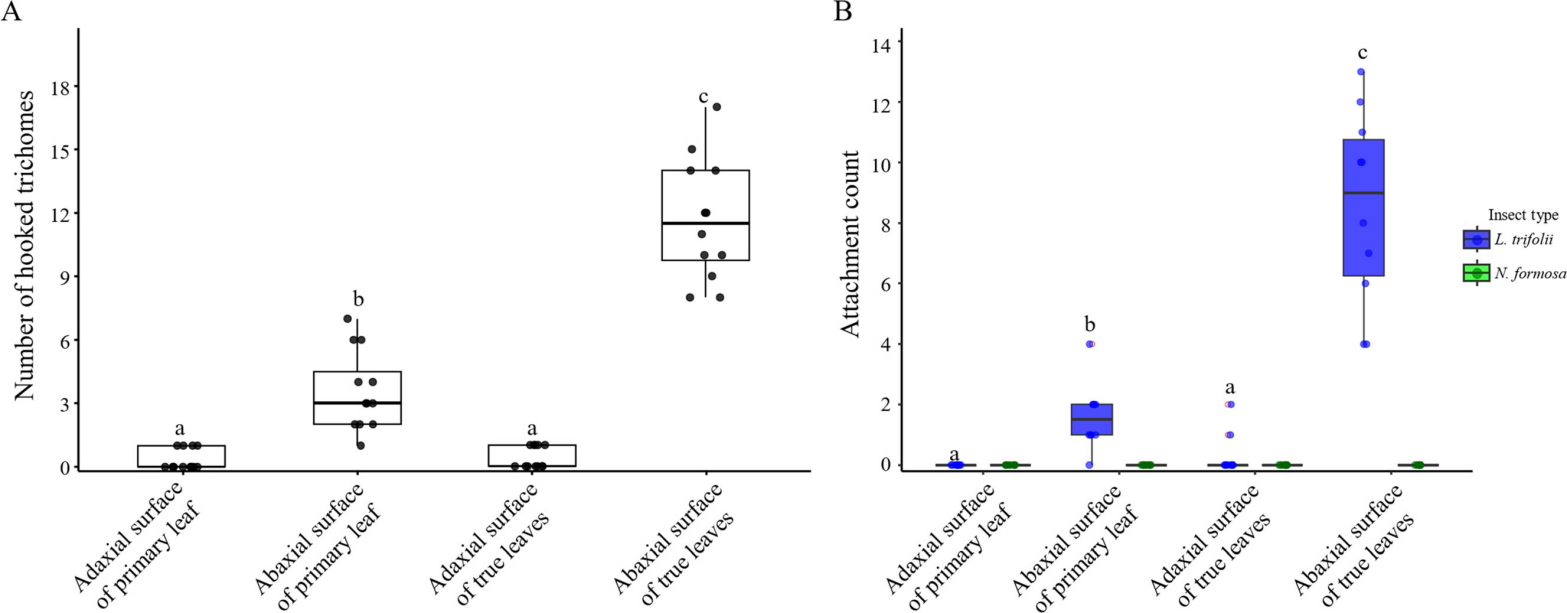
Relationship between trichome density and attachment behavior of *Liriomyza trifolii*. Each panel presents box plots with jittered individual data points (*n* = 10 replicates per condition). Boxes indicate medians and interquartile ranges; red dots represent outliers. (A) Trichome density across different plant parts. Different letters indicate significant differences (Poisson generalized liner mixed models (GLMM) with Tukey's honestly significant difference (HSD) test, *P* < 0.05). (B) Number of individuals attached to each plant part. Blue, *L. trifolii*; green, *Neochrysocharis formosa*. Different letters indicate significant differences (*L. trifolii* only; negative binomial regression with Bonferroni correction, *P* < 0.05). No statistical test was performed for *N. formosa* due to zero counts.

SEM revealed the presence of various types of trichomes—hook‐shaped, needle‐shaped, and small glandular—on both adaxial and abaxial surfaces of kidney bean leaves (Fig. 1(A)–(D)). Notably, trichomes were more densely distributed on the abaxial surface of true leaves compared to other leaf regions (Fig. 1(D); see also Supporting Information, Fig. S1 for magnified hook‐shaped trichomes). Quantitative analysis using a Poisson GLMM revealed significant differences in trichome density across leaf types and surfaces (*P* < 0.05; Fig. 2(A)).

*Post hoc* Tukey's HSD tests indicated no significant difference between the adaxial surfaces of cotyledons and true leaves (*P* = 0.987). In contrast, the abaxial surface of true leaves had significantly higher trichome density than that of cotyledons (*P* < 0.001). In both leaf types, abaxial surfaces exhibited significantly higher densities than adaxial surfaces (*P* < 0.001), with the abaxial surface of true leaves showing the highest overall density.

To evaluate whether these structural differences influence insect attachment patterns, attachment counts of *L. trifolii* on the four leaf surfaces were analyzed using negative binomial regression (Fig. 2(B)). The analysis revealed a significant effect of leaf position on attachment frequency (*P* < 0.001), suggesting that variation in trichome distribution physically restricts *L. trifolii* movement and contributes to differential trapping patterns. *Post hoc* comparisons (Bonferroni‐adjusted) showed that the abaxial surface of true leaves exhibited significantly higher attachment than all other surfaces (*P* < 0.05), including the adaxial surface of true leaves (estimated mean = 0.3), the abaxial surface of cotyledons (1.6), and the adaxial surface of cotyledons, which showed no attachment (estimated mean = 0.0). In contrast, *N. formosa* showed no attachment behavior on any of the tested leaf surfaces in any replicate. This consistent absence of contact under experimental conditions clearly indicates that this parasitoid species scarcely interacts with the host plant surface. Because attachment counts were uniformly zero, statistical comparisons between *L. trifolii* and *N. formosa* could not be performed.

Finally, neither sex nor age (days post‐eclosion) significantly affected the likelihood of being trapped on the leaf surface by trichomes in *L. trifolii* (Supporting Information, Fig. S2). Of the 300 individuals tested per sex, 120 females (40.0%) and 115 males (38.3%) were trapped, with no significant difference between sexes (*χ* ^2^ = 0.11, *P* = 0.74). These results suggest that the entrapment effect is consistent regardless of these biological factors. Representative images and a video showing *L. trifolii* individuals trapped by trichomes are provided in Supporting Information, Fig. S3 and Video S1.

### 3.2 Effect of trichome removal on attachment and oviposition of *L. trifolii*

**Figure 3:**
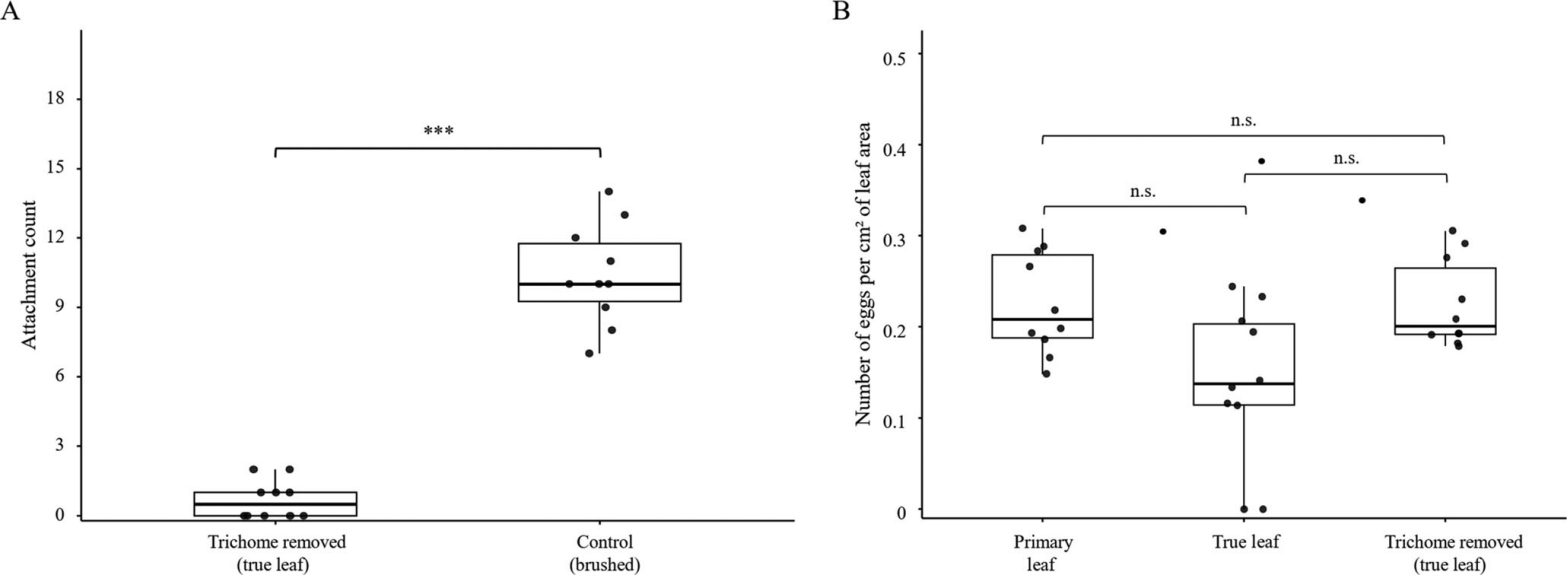
Effects of trichome removal on *Liriomyza trifolii* attachment and oviposition. Each panel presents box plots with jittered individual data points (*n* = 10 replicates per treatment). Boxes represent medians and interquartile ranges. In each replicate, 30 adults *L. trifolii* individuals were released (total *n* = 300 per treatment). (A) Attachment rates of *L. trifolii* adults on the abaxial surface of leaves under two treatments: control (mechanical stimulation) and trichome removal. Asterisks indicate significant differences between treatments (Wilcoxon test, *P* < 0.05). (B) Number of eggs laid by *L. trifolii* under the same treatments. n.s., no significant difference (Kruskal–Wallis test, *P* = 0.053).

To evaluate the role of trichomes in facilitating attachment, we compared the number of *L. trifolii* individuals trapped on the adaxial surface of true leaves with or without trichomes. In the control group, where leaves were brushed but trichomes remained intact, an average of 10.4 ± 2.0 individuals were found attached. In contrast, in the trichome‐removal group, attachment dropped dramatically to 0.8 ± 0.8 individuals on average, with no individuals observed in six out of 10 replicates (Fig. 3(A)).

A negative binomial regression analysis confirmed that attachment was significantly reduced in the trichome‐removal group compared to the control (estimate = 2.70, standard error = 0.39, *z* = 6.91, *P* < 0.001).

Oviposition assays provided additional support for the role of trichomes. The number of oviposition punctures on trichome‐removed true leaves was comparable to that on cotyledons, which naturally exhibit low trichome densities (Fig. 3(B)). Although the Kruskal–Wallis test revealed only a marginally non‐significant difference in oviposition density among the three treatments (*χ* ^2^ = 5.87, df = 2, *P* = 0.053), the observed pattern suggests that the structural characteristics of the leaf surface may influence *L. trifolii*'s oviposition behavior.

Taken together, these results indicate that the presence of trichomes substantially increases the likelihood of physical entrapment and may contribute to a marked suppression of oviposition activity in *L. trifolii*.

### 3.3 Effect of trichomes on parasitoid efficiency of *N. formosa*

**Figure 4:**
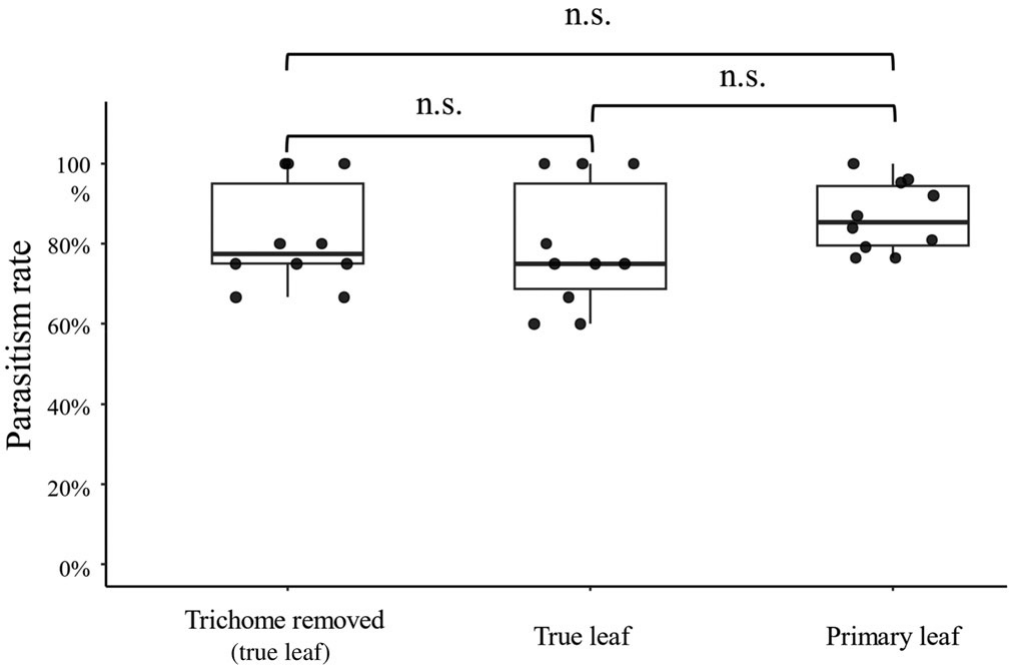
Parasitism rate of *Neochrysocharis formosa* on kidney bean leaves with and without trichomes. Comparisons were made among trichome‐removed true leaves, intact true leaves, and intact primary leaves (*n* = 10 per treatment). Parasitism rate was calculated as the number of parasitoid mummies divided by the initial number of *Liriomyza trifolii* larvae. Boxplots indicate medians and interquartile ranges, with dots representing individual data points. No significant differences were detected among treatments (Kruskal–Wallis test, *P* = 0.29). n.s., no significant difference.

To verify whether trichomes hinder the functional efficiency of natural enemies, we compared the parasitism rates of *N. formosa* among three treatments: trichome‐removed true leaves, untreated true leaves, and untreated primary leaves. High parasitism rates were maintained across all treatments (approximately 80–87%; Fig 4) and no significant differences were observed (Kruskal–Wallis test, *χ* ^2^ = 2.48, df = 2, *P* = 0.29). The mean parasitism rates were 81.8% for trichome‐removed leaves, 79.2% for intact true leaves, and 86.7% for intact primary leaves, respectively. These results indicate that the presence of trichomes does not impede the parasitoid's ability to locate and attack hosts.

### 3.4 Species‐specific differences in trichome‐mediated attachment

To assess whether trichome‐mediated entrapment varies across insect taxa, we compared attachment rates among multiple insect species released onto kidney bean plants.

**Figure 5:**
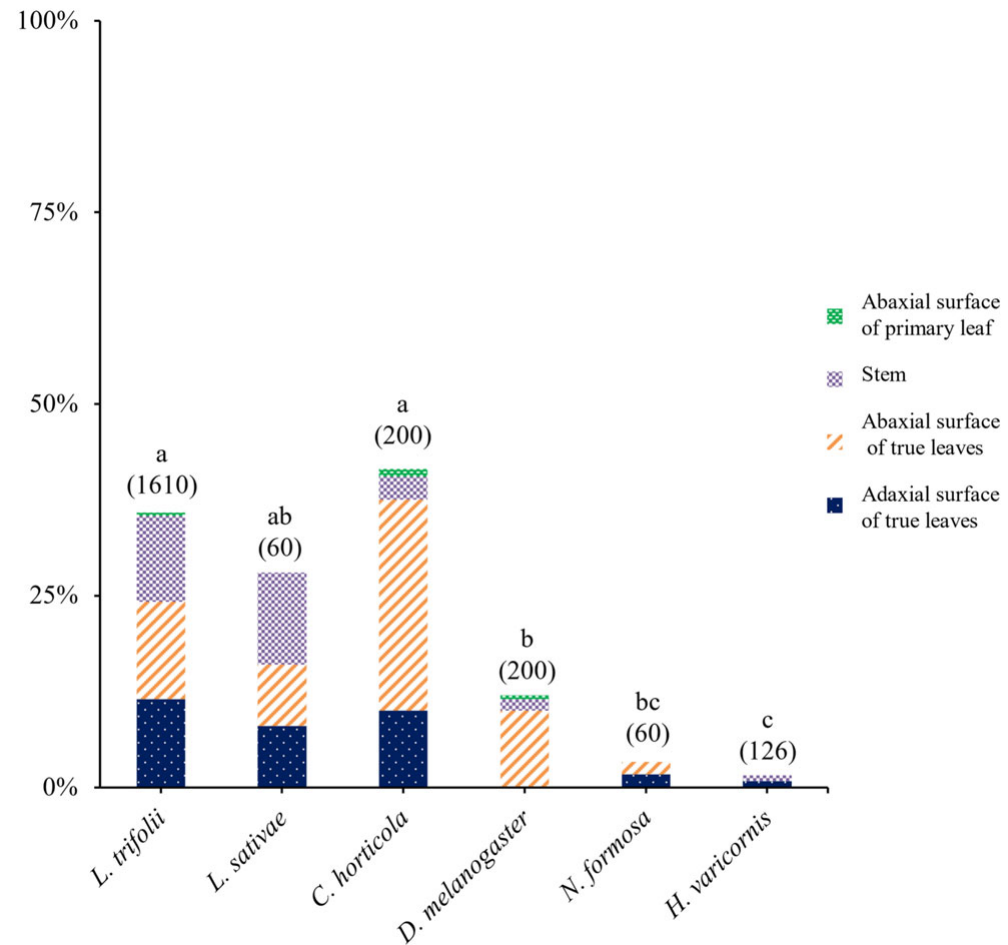
Species‐specific differences in trichome‐mediated attachment across plant surfaces. The percentage of individuals of each insect species found attached to kidney bean surfaces 24 h after release is shown. Bars represent proportions of attachment on four plant regions: the abaxial surface of primary leaf, the stem, the abaxial surface of true leaves, and the adaxial surface of true leaves. Sample sizes (number of individuals tested) are indicated in parentheses. As shown in the figure, attachment rates varied significantly among species (*χ* ^2^ test with Ryan's multiple comparison, *P* < 0.05). Different lowercase letters indicate statistically significant differences.

As shown in Fig. 5, attachment rates varied significantly among species (*χ* ^2^ test with Ryan's multiple comparison). The leafminers *C. horticola* and *L. trifolii* showed the highest attachment rates, at 41.5% (*n* = 200) and 35.8% (*n* = 1610), respectively, both significantly higher than those of the parasitoid wasps and the unrelated species. *L. sativae* had a moderately high attachment rate of 28.0% (*n* = 60), which was statistically intermediate.

In contrast, parasitoid wasps showed markedly lower attachment rates: *N. formosa* at 3.3% (*n* = 60) and *H. varicornis* at 1.6% (*n* = 126), both significantly lower than those of all leafminer species. *D. melanogaster*, an ecologically unrelated species, showed an intermediate rate of 12.0% (*n* = 200), which was higher than parasitoids but lower than leafminers.

Detailed analysis of 570 entrapped *L. trifolii* individuals revealed that entrapment frequently involved multiple body parts: 18.2% were captured by both legs and ovipositor, and 21.0% by legs and proboscis (Supporting Information, Fig. S4A–C). The specific entrapment of the ovipositor suggests active attempts to probe or oviposit at the site of entrapment. Notably, actual oviposition punctures on the abaxial surface were observed only in *C. horticola* (Supporting Information, Fig. S4D), which is consistent with this species' ecological preference for abaxial oviposition. In contrast, despite the high rate of ovipositor entrapment, no successful punctures were found for *L. trifolii*. This suggests that while active probing increases the risk of entanglement, trichomes effectively intercept *L. trifolii* females before they can successfully penetrate the tissue.

### 3.5 Inter‐ and intraspecific differences in leg thickness

**Figure 6:**
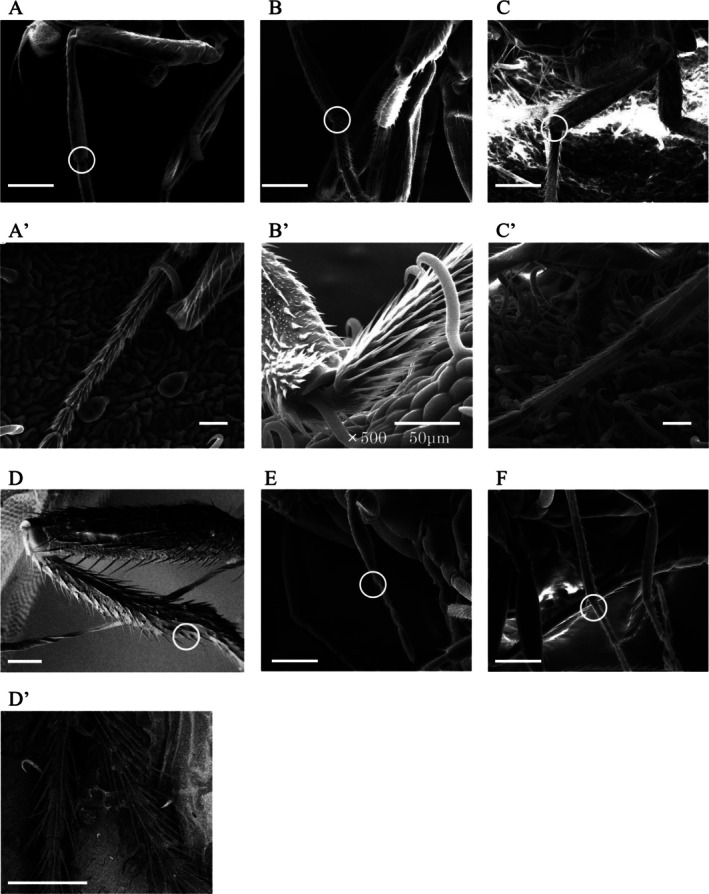
Representative scanning electron microscopy images of insect hind legs and their entrapment on kidney bean trichomes. Images with primes (A′–D′) correspond to the tibia–tarsus junction region indicated by yellow circles in the unprimed panels (A–D). They are independent images of trichome entrapment, not simple enlargements or crops of A–D. All images except for B′ were taken at ×150 magnification, with 100 μm scale bars unless otherwise indicated. (A) Hind leg of *Liriomyza trifolii* (leafminer). (A') *L. trifolii* leg entrapped by trichomes on the abaxial surface of a kidney bean leaf. (B) Hind leg of *L. sativae* (leafminer). (B′) *L. sativae* leg entrapped by trichomes. A scale bar and magnification (×500, 50 μm) are shown. (C) Hind leg of *Chromatomyia horticola*. (C′) *C. horticola* leg entangled in trichomes. (D) Hind leg of *Drosophila melanogaster* (fruit fly). (D′) *D. melanogaster* leg entangled in trichomes. (E) Hind leg of *Neochrysocharis formosa* (parasitoid wasp). (F) Hind leg of *Hemiptarsenus varicornis* (another parasitoid wasp).

To explore potential morphological factors underlying the observed species‐specific differences in trichome‐mediated attachment, we compared leg morphology across insect species using SEM. These differences in attachment susceptibility appeared consistent with structural variation in leg surface features (Fig. 6). The three leafminer species that exhibited high attachment rates—*L. trifolii*, *L. sativae*, and *C. horticola*—shared similar leg morphologies characterized by numerous fine hairs and textured cuticle surfaces that appeared prone to entanglement with trichomes (Fig. 6(A)–(C)).

In contrast, the legs of *D. melanogaster* and the parasitoid wasps *N. formosa* and *H. varicornis* exhibited distinct structures, with apparently fewer or differently arranged setae (Fig. 6(D)–(F)), although these traits were not quantitatively measured. These morphological distinctions may contribute to their reduced susceptibility to trichome‐induced entrapment. Notably, close‐up images revealed that trichomes physically intruded into the tibia–tarsus joint or adhered to the leg surface in leafminers (Fig. 6(A′)–(C′)), providing visual evidence of mechanical entrapment facilitated by leg morphology.

**Figure 7:**
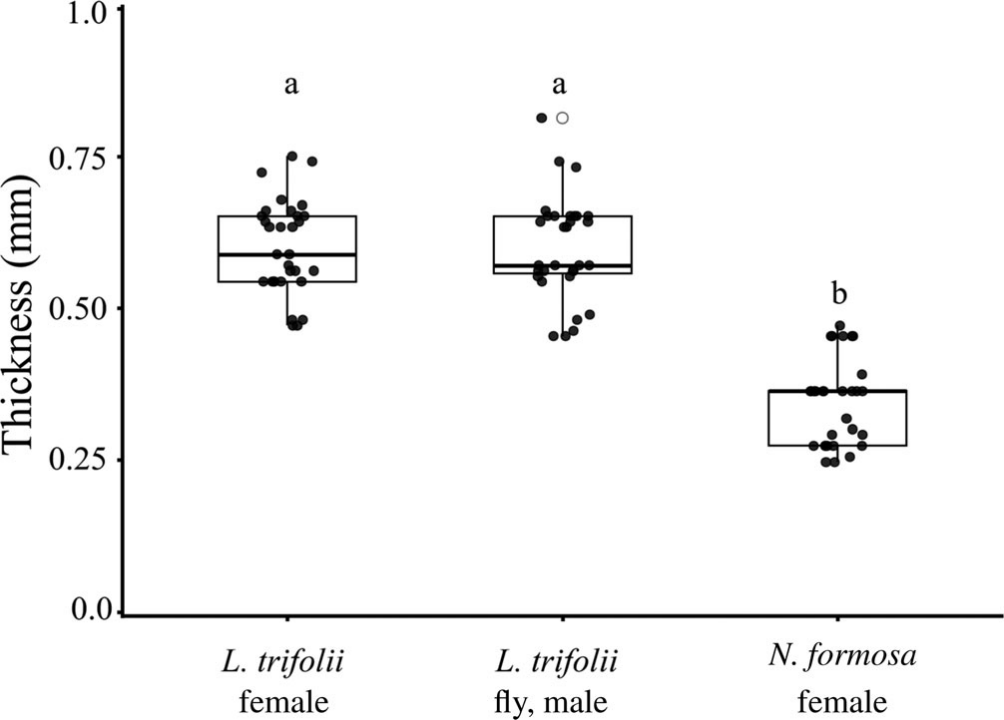
Hind tibia width of *Liriomyza trifolii* and *Neochrysocharis formosa*. Hind tibia widths were measured for female and male *L. trifolii* and female *N. formosa*. Each point represents an individual measurement, with boxplots indicating medians and interquartile ranges; outliers are shown as white circles with red outlines. Significant differences were found among groups (Kruskal–Wallis test, *P* < 0.001), with *N. formosa* exhibiting significantly thinner tibiae than both sexes of *L. trifolii*. No significant difference was detected between male and female *L. trifolii*. Different letters above boxes indicate significant differences (Dunn's *post hoc* test, *P* < 0.05).

To further investigate whether leg thickness contributes to variation in attachment, we measured hind tibia thickness in *L. trifolii* females and males, and in *N. formosa* females (Fig. 7). Dunn's multiple comparison test with Bonferroni correction revealed no significant difference between the sexes of *L. trifolii* (*P* = 1.000), but *N. formosa* had significantly thinner tibiae than both female and male *L. trifolii* (*P* < 0.001). These results suggest that thinner leg structures in *N. formosa* may reduce the likelihood of physical entrapment by trichomes, complementing the qualitative morphological observations.

## 4 DISCUSSION

This study demonstrates that trichomes on kidney bean plants exhibit strong species‐specific effects on insect attachment and oviposition behaviors, mediated in part by morphological differences such as leg thickness. Importantly, these effects need to be interpreted in the context of insect life stages: while adults of *Liriomyza* spp. feed by probing and ovipositing mainly on the adaxial surface of leaves, their larvae damage plants by mining leaf tissues. Our findings highlight the potential of trichomes to serve as selective physical barriers, entrapping herbivorous pests while sparing their natural enemies.

Consistent with prior reports that trichomes function as mechanical defenses against herbivory,^34^ we observed significantly higher trichome densities on the abaxial surfaces of true leaves, where *L. trifolii* adults exhibited the highest attachment rates. Interestingly, attachment patterns were not influenced by sex or age (days post‐eclosion), suggesting that the observed behavioral differences are governed by external structural cues rather than intrinsic biological variables.

When trichomes were experimentally removed from the surface of kidney bean leaves, the attachment of *L. trifolii* adults significantly decreased, whereas their oviposition frequency showed an increasing trend. This suggests that while trichomes promote physical adherence to the leaf surface, they may also act as deterrents to oviposition under natural conditions. A small number of flies occasionally adhered even on trichome‐removed leaves, most likely due to residual trichome bases or partially removed trichomes that still provided anchoring points for the leafminer legs. Importantly, because *L. trifolii* typically oviposits and feeds on the adaxial surface, the ecological relevance of abaxial trichome density may relate more to restricting adult locomotion and probing than to directly blocking oviposition. Although oviposition density on a single leaf did not differ significantly between treatments, the ecological impact in a field setting—where kidney bean plants possess dense foliage—is likely substantial. In our single‐leaf assays, flies were forced to interact with a specific leaf surface. However, under natural conditions, adult flies typically move between multiple leaves to maximize oviposition. The entrapment of an adult on one leaf effectively terminates its reproductive lifespan, preventing it from ovipositing on subsequent leaves. Therefore, by functioning as a ‘lethal sink’ that causes pre‐reproductive mortality, trichomes likely act as an indirect barrier that protects the entire plant from larval infestation, even if they do not physically prevent oviposition on the immediate surface.

In contrast, the parasitoid *N. formosa* displayed negligible attachment across all plant surfaces, despite being ecologically associated with *Liriomyza* hosts. Comparative tibia measurements showed that *N. formosa* possesses significantly thinner legs than *L. trifolii*, regardless of sex. Because insect legs are not perfectly cylindrical but often elliptical in cross‐section, our width measurements provide an approximation; however, the consistency across large sample sizes strengthens the conclusion that leg slenderness reduces mechanical entanglement. This interpretation is supported by earlier observations in predatory bugs [e.g., *Pameridea roridulae* (Reuter)], where elongated, slim legs enable movement across glandular surfaces.^35^ These results suggest that parasitoids may have evolved physical adaptations for trichome‐rich environments, allowing them to exploit hosts without being hindered by plant defenses.

Furthermore, species‐level comparisons revealed that herbivorous leafminers (*L. trifolii*, *L. sativae*, and *C. horticola*) experienced substantially higher attachment rates than either parasitoid wasps or an ecologically unrelated species (*D. melanogaster*), indicating that trichome entrapment is governed primarily by physical interaction rather than ecological association. The moderate attachment observed in *D. melanogaster*, a species that does not naturally associate with kidney bean, further supports the view that leg morphology—rather than trophic role—determines susceptibility.

While leg thickness appears to be an important determinant of entrapment, it is unlikely to be the sole factor. Although our data suggest that thinner leg structures in parasitoids may contribute to their lower entrapment rates, this relationship currently remains correlational. Similar vulnerability of small‐bodied insects to trichome‐based defenses has been documented in other systems, for example in aphids on *Nicotiana* species and in various small herbivores on tomato and wild *Solanum* relatives.^36^, ^37^, ^38^, ^39^ Therefore, larger parasitoid species, or those with specialized tarsal adaptations, could interact with trichomes quite differently from the small‐bodied species examined here. In addition, behavioral agility or cuticular surface chemistry may also influence outcomes.^40^, ^41^ Future work involving biomechanical measurements or morphological manipulation is required to experimentally isolate these factors from functional identity.

Moreover, kidney bean fields host a range of pests beyond leafminers, including aphids and mites, which may also be affected by trichomes. Understanding how diverse insects respond to these structures will require broader comparative studies. At the same time, it remains unclear whether *L. trifolii* has evolved specific traits to overcome the oviposition‐inhibiting effects of trichomes; investigations into oviposition site selection, avoidance behaviors, or morphological modifications may reveal coevolutionary dynamics between herbivores and plant surface traits.

The ecological implications of these findings are twofold. First, from a plant defense perspective, trichomes may offer selective protection that suppresses herbivorous pests while minimizing interference with beneficial parasitoids. Previous work^42^ showed that increased trichome density in kidney bean reduced the longevity of *L. trifolii*, suggesting that trichomes can impose substantial fitness costs on leafminers. Together with our evidence that trichomes impede adult mobility, these findings indicate that trichomes act through multiple defensive pathways. This perspective also aligns with studies in cotton reporting that high trichome density can reduce parasitism rates,^26^ raising concerns about trade‐offs between direct and indirect defenses.^43^ However, our trichome‐removal experiment explicitly demonstrated that such a trade‐off is absent in this system. Parasitism rates of *N. formosa* remained consistently high (approximately 80–87%) regardless of the presence of trichomes, confirming that these physical structures do not impede the parasitoid's host‐searching or oviposition efficiency. Consequently, trichome structure in kidney bean creates an asymmetric barrier—impeding pest activity while sparing natural enemies. Second, from an applied perspective, our findings provide a mechanistic basis for developing pest‐resistant cultivars that support biological control. Since commercial production typically relies on plants with multiple true leaves rather than cotyledons, trichome traits on true leaves are particularly relevant for crop protection. Although rigorous field testing is required to confirm practical efficacy under complex environmental conditions, the potential to engineer or breed plants with trichomes optimized for pest exclusion but parasitoid compatibility represents a promising avenue for sustainable pest management.

In conclusion, this study provides mechanistic insights into how trichome morphology and insect leg structure interact to produce species‐specific attachment outcomes. By integrating behavioral assays, morphological measurements, and interspecific comparisons, we show that—at least under laboratory conditions—trichome‐mediated defense can function as a targeted barrier against pests without disrupting the ecological services provided by their natural enemies. Validating whether these patterns translate to effective pest suppression in the field remains a critical next step. Future work should investigate whether similar patterns hold in field conditions and across a wider range of crop systems and trichome types.

## 5 CONCLUSION

This study reveals that trichomes on kidney bean leaves can function as selective physical barriers, disproportionately entrapping herbivorous leafminers while sparing beneficial parasitoid wasps. This species‐specific defense is primarily mediated by differences in leg morphology, with slender‐legged parasitoids avoiding entrapment. The findings highlight a potential trade‐off resolution in plant defense: physical structures that deter pests without hindering natural enemies. Such selective interactions between plant morphology and insect traits offer promising avenues for breeding pest‐resistant crops compatible with biological control strategies.

## CONFLICT OF INTEREST

All authors declare no conflict of interest.

## AUTHOR CONTRIBUTIONS

YO, YK, and YT conceived the study and designed the experiments. YO and YS conducted the experiments and performed statistical analyses. YS and YI contributed to methodology and data interpretation. YS and YT provided access to resources. YO prepared the figures and drafted the manuscript. All authors contributed to manuscript revisions and approved the final version.

## Supporting information


**Figure S1.** Hook‐shaped trichomes on the abaxial surface of a true leaf. Hook‐shaped trichomes are densely distributed and oriented towards the leaf surface. Scale bar: 50 μm.
**Figure S2.** Effects of age on the trichome‐mediated attachment in *Liriomyza trifolii*. Attachment (adhesion) ratios of adults measured on each day from 1 to 8 days post‐eclosion. No significant trend in attachment was observed across ages (*χ* ^2^ test, *P* = 0.7303). The average attachment rate remained approximately 15% throughout.
**Figure S3.** *Liriomyza trifolii* individuals trapped by leaf trichomes. (A) Individual with legs and mouthparts entrapped and deceased. (B) Individual with the lateral side of a leg entrapped. (C) Deceased individual with legs entrapped and desiccated. (D) Living individual immediately after leg entrapment.
**Figure S4.** Detailed analysis of trichome entrapment modes in leafminers. (A) Breakdown of trapped body parts of *Liriomyza trifolii* on the abaxial surfaces of true leaves, primary leaves, and stems. Stacked bars represent the number of individuals trapped by legs only (orange), legs and ovipositor (blue), legs and proboscis (green), or other parts (gray). Data correspond to the species‐specific attachment analysis shown in Fig. 5. (B) Representative stereomicroscope image of an *L. trifolii* adult with its proboscis (mouthpart) entrapped by a hook‐shaped trichome. The white scale bar represents 100 μm. (C) Close‐up of an *L. trifolii* female with her ovipositor entrapped by trichomes. The white scale bar represents 100 μm. (D) Oviposition punctures created by *Chromatomyia horticola* on the abaxial surface of a kidney bean plant leaf. The white scale bar represents 100 μm.

**Video S1.** A live *Liriomyza trifolii* individual attempting to escape after its leg is caught by a hook‐shaped trichome on the abaxial surface of a true leaf.

## Data Availability

The data that support the findings of this study are available from the corresponding author upon reasonable request.

## References

[1] Wagner GJ , Wang E and Shepherd RW , New approaches for studying and exploiting an old protuberance, the plant trichome. Ann Bot 93:3–11 (2004). 14678941 10.1093/aob/mch011 PMC4242265

[2] Werker E , Trichome diversity and development, in Advances in Botanical Research. vol. 31, Academic press, New York, USA, pp. 1–35 (2000).

[3] Levin DA , The role of trichomes in plant defense. Q Rev Biol 48:3–15 (1973).

[4] Handley R , Ekbom B and Agren J , Variation in trichome density and resistance against a specialist insect herbivore in natural populations of *Arabidopsis thaliana* . Ecol Entomol 30:284–292 (2005).

[5] Spencer KA , Division BRYOPHYTA, in Host Specialization in the World Agromyzidae (Diptera). Springer Netherlands, Dordrecht, pp. 1–3 (1990).

[6] Saito T , Oishi T and Ikeda F , Resurgence of *Liriomyza trifolii* Burgess caused by the application of permethrin in a greenhouse Proc Kanto‐Tosan Plant Prot Soc, p. 40 (1993).

[7] Iwasaki A , A newly recorded pest, *Liriomyza sativae* Blanchard in Japan. Plant Prot 54:12–17 (2000).

[8] Xing Z , Liu Y , Cai W , Huang X , Wu S and Lei Z , Efficiency of trichome‐based plant defense in *Phaseolus vulgaris* depends on insect behavior, plant ontogeny, and structure. Front Plant Sci 8:2006 (2017). 29225609 10.3389/fpls.2017.02006 PMC5705610

[9] Liu TX , Kang Le KL , Heinz KM and Trumble J , Biological control of *Liriomyza* leafminers: progress and perspective. CAB Rev Perspect Agric Vet Sci Nutr Nat Resour 4:1–16 (2009).

[10] Ridland PM , Umina PA , Pirtle EI and Hoffmann AA , Potential for biological control of the vegetable leafminer, *Liriomyza sativae* (Diptera: Agromyzidae), in Australia with parasitoid wasps. Aust Entomol 59:16–36 (2020).

[11] Tagami Y , Doi M , Sugiyama K , Tatara A and Saito T , Survey of leafminers and their parasitoids to find endosymbionts for improvement of biological control. Biol Control 38:210–216 (2006).

[12] Wu Y and Abe Y , Egg maturation and daily progeny production in the parasitoid, *Gronotoma micromorpha* (Hymenoptera: Figitidae: Eucoilinae). J Econ Entomol 113:2546–2548 (2020). 32609363 10.1093/jee/toaa146

[13] Kemmochi T , Fujimori S and Saito T , The leafminer *Liriomyza trifolii* (Diptera: Agromyzidae) encapsulates its koinobiont parasitoid *Halticoptera circulus* (Hymenoptera: Pteromalidae): implications for biological control. Bull Entomol Res 106:322–327 (2016). 26639841 10.1017/S0007485315000930

[14] Konishi K , Illustrations search for leafminer parasitoids. Nogyokankyogijutukenkyujosiryo 22:27–76 (1998).

[15] Tokumaru S and Abe Y , Hymenopterous parasitoids of leafminers, *Liriomyza sativae* Blanchard, L. *trifolii* (Burgess), and *L. bryoniae* (Kaltenbach) in Kyoto prefecture. Jpn J Appl Entomol Zool 50:341–345 (2006).

[16] Amano K , Suzuki A , Hiromori H and Saito T , Relative abundance of parasitoids reared during field exposure of sentinel larvae of the leafminers *Liriomyza trifolii* (Burgess), *L. sativae* Blanchard, and *Chromatomyia horticola* (Goureau) (Diptera: Agromyzidae). Appl Entomol Zool 43:625–630 (2008).

[17] Saito T , Doi M , Tagami Y and Sugiyama K , Hymenopterous parasitoids of the exotic leafminers *Liriomyza trifolii* (burgess) and *Liriomyza sativae* Blanchard (Diptera: Agromyzidae) in Shizuoka prefecture, Japan. Jpn J Appl Entomol Zool 52:225–229 (2008).

[18] Cheng X‐Q , Cao F‐Q , Zhang Y‐B , Guo J‐Y , Wan F‐H and Liu W‐X , Life history and life table of the host‐feeding parasitoid *Hemiptarsenus varicornis* (Hymenoptera: Eulophidae). Appl Entomol Zool 52:287–293 (2017).

[19] Osmankhil MH , Mochizuki A , Hamasaki K and Iwabuchi K , Oviposition and larval development of *Neochrysocharis formosa* (Hymenoptera: Eulophidae) inside the host larvae, Liriomyza trifolii. Japan Agri Res Quarterly 44:33–36 (2010).

[20] Tran DH , Takagi M and Takasu K , Effects of selective insecticides on host searching and oviposition behavior of *Neochrysocharis formosa* (Westwood) (Hymenoptera: Eulophidae), a larval parasitoid of the American serpentine leafminer. Appl Entomol Zool 39:435–441 (2004).

[21] Bethke JA and Parrella MP , Leaf puncturing, feeding and oviposition behavior of *Liriomyza trifolii* . Entomol Exp Appl 39:149–154 (1985).

[22] Tian D , Tooker J , Peiffer M , Chung SH and Felton GW , Role of trichomes in defense against herbivores: comparison of herbivore response to woolly and hairless trichome mutants in tomato (*Solanum lycopersicum*). Planta 236:1053–1066 (2012). 22552638 10.1007/s00425-012-1651-9

[23] War AR , Paulraj MG , Ahmad T , Buhroo AA , Hussain B , Ignacimuthu S *et al*., Mechanisms of plant defense against insect herbivores. Plant Signal Behav 7:1306–1320 (2012). 22895106 10.4161/psb.21663 PMC3493419

[24] Kennedy GG , Tomato, pests, parasitoids, and predators: tritrophic interactions involving the genus Lycopersicon. Annu Rev Entomol 48:51–72 (2003). 12194909 10.1146/annurev.ento.48.091801.112733

[25] Riddick EW and Simmons AM , Do plant trichomes cause more harm than good to predatory insects? Pest Manag Sci 70:1655–1665 (2014). 24585676 10.1002/ps.3772

[26] Treacy MF , Benedict JH , Segers JC , Morrison RK and Lopez JD , Role of cotton trichome density in bollworm (Lepidoptera: Noctuidae) egg parasitism. Environ Entomol 15:365–368 (1986).

[27] Heil M , Indirect defence via tritrophic interactions. New Phytol 178:41–61 (2008). 18086230 10.1111/j.1469-8137.2007.02330.x

[28] Frank SD , Biological control of arthropod pests using banker plant systems: past progress and future directions. Biol Control 52:8–16 (2010).

[29] Ohata Y and Tagami Y , Antibiotic agrochemical treatment reduces endosymbiont infections and alters population dynamics in leafminers, thrips, and parasitoid wasps. Front Microbiol 16:1605308 (2025). 40556890 10.3389/fmicb.2025.1605308 PMC12185494

[30] Adachi‐Hagimori T and Miura K , Development of a multiplex method to discriminate between *Neochrysocharis formosa* (Hymenoptera: Eulophidae) reproductive modes. J Econ Entomol 101:1510–1514 (2008). 18767766 10.1603/0022-0493(2008)101[1510:doammt]2.0.co;2

[31] Hagimori T , Abe Y , Date S and Miura K , The first finding of a rickettsia bacterium associated with parthenogenesis induction among insects. Curr Microbiol 52:97–101 (2006). 16450063 10.1007/s00284-005-0092-0

[32] R: A language and environment for statistical computing. R Foundation for Statistical Computing, Vienna (2024).

[33] Ryan TA , Significance tests for multiple comparison of proportions, variances, and other statistics. Psychol Bull 57:318–328 (1960). 14440422 10.1037/h0044320

[34] Fukuda T , Sakamaki Y , Kamiwada H and Kusigemati K , Host preference of *Liriomyza trifolii* (Burgess) among some leguminous crops originated from Kagoshima. Bull Exp Farm Fac Agr Kagoshima Univ 26:1–10 (2001).

[35] Voigt D and Gorb S , Locomotion in a sticky terrain. Arthropod Plant Interact 4:69–79 (2010).

[36] Glas JJ , Schimmel BCJ , Alba JM , Escobar‐Bravo R , Schuurink RC and Kant MR , Plant glandular trichomes as targets for breeding or engineering of resistance to herbivores. Int J Mol Sci 13:17077–17103 (2012). 23235331 10.3390/ijms131217077 PMC3546740

[37] Zhang Y , Song H , Wang X , Zhou X , Zhang K , Chen X *et al*., The roles of different types of trichomes in tomato resistance to cold, drought, whiteflies, and botrytis. Agronomy (Basel) 10:411 (2020).

[38] Simmons AT , Gurr GM , McGrath D , Martin PM and Nicol HI , Entrapment of *Helicoverpa armigera* (Hübner) (Lepidoptera: Noctuidae) on glandular trichomes of *Lycopersicon* species. Aust J Entomol 43:196–200 (2004).

[39] Wang E , Hall JT and Wagner GJ , Transgenic *Nicotiana tabacum* L. with enhanced trichome exudate cembratrieneols has reduced aphid infestation in the field. Mol Breed 13:49–57 (2004).

[40] Eigenbrode SD and Espelie KE , Effects of plant epicuticular lipids on insect herbivores. Annu Rev Entomol 40:171–194 (1995).

[41] Hanley ME , Lamont BB , Fairbanks MM and Rafferty CM , Plant structural traits and their role in anti‐herbivore defence. Perspect Plant Ecol Evol Syst 8:157–178 (2007).

[42] QulRing DT , Timmins PR and Park SJ , Effect of variations in hooked trichome densities of *Phaseolus vulgaris* on longevity of *Liriomyza trifolii* (Diptera: Agromyzidae) adults. Environ Entomol 21:1357–1361 (1992).

[43] Gassmann AJ and Hare JD , Indirect cost of a defensive trait: variation in trichome type affects the natural enemies of herbivorous insects on *Datura wrightii* . Oecologia 144:62–71 (2005). 15800744 10.1007/s00442-005-0038-z

