# Supplementary material for: Selective leaf surface defenses: trichomes trap herbivorous leafminers but spare parasitoid wasps
Source: Pest Manag Sci. 2026 Jan 15;82(4):4016–25. doi: 10.1002/ps.70523 (PMC12976177; doi:10.1002/ps.70523)
Supplement: Supplementary file 1 — Figure S1. Hook‐shaped trichomes on the abaxial surface of a true leaf. Hook‐shaped trichomes are densely distributed and oriented towards the leaf surface. Scale bar: 50 μm. Figure S2. Effects of age on the trichome‐mediated attachment in Liriomyza trifolii. Attachment (adhesion) ratios of adults measured on each day from 1 to 8 days post‐eclosion. No significant trend in attachment was observed across ages (χ 2 test, P = 0.7303). The average attachment rate remained approximately 15% throughout. Figure S3. Liriomyza trifolii individuals trapped by leaf trichomes. (A) Individual with legs and mouthparts entrapped and deceased. (B) Individual with the lateral side of a leg entrapped. (C) Deceased individual with legs entrapped and desiccated. (D) Living individual immediately after leg entrapment. Figure S4. Detailed analysis of trichome entrapment modes in leafminers. (A) Breakdown of trapped body parts of Liriomyza trifolii on the abaxial surfaces of true leaves, primary leaves, and stems. Stacked bars represent the number of individuals trapped by legs only (orange), legs and ovipositor (blue), legs and proboscis (green), or other parts (gray). Data correspond to the species‐specific attachment analysis shown in Fig. 5. (B) Representative stereomicroscope image of an L. trifolii adult with its proboscis (mouthpart) entrapped by a hook‐shaped trichome. The white scale bar represents 100 μm. (C) Close‐up of an L. trifolii female with her ovipositor entrapped by trichomes. The white scale bar represents 100 μm. (D) Oviposition punctures created by Chromatomyia horticola on the abaxial surface of a kidney bean plant leaf. The white scale bar represents 100 μm. [file PS-82-4016-s002.docx]

**Supplementary material**

**Selective leaf trichome defenses: trapping invasive leafminers but sparing parasitoid wasps on kidney bean leaves**

**Author names and affiliations:**

Yuta Ohata^a^*, Yuhko Sawada^†^, Yuki Ishihara^b†^, Yohsuke Tagami^c^*

*^a^* Gifu University, United Graduate School of Agricultural Science, Gifu, Japan

*^b^* Fisheries Resources Institute, Japan Fisheries Research and Education Agency, Yokohama,Japan

^c^ Shizuoka University, Faculty of Agriculture, Shizuoka, Japan

†Y.S. and Y.I. conducted this work while affiliated with the Shizuoka University, Faculty of Agriculture, Shizuoka, Japan.

**Correspondence*: [ohata.yuta@gmail.com](mailto:ohata.yuta@gmail.com), [tagamiy@gmail.com](file:///C:\Users\siobh\Downloads\もうじedit\tagamiy@gmail.com) Tel & Fax: +81(54)238-4825


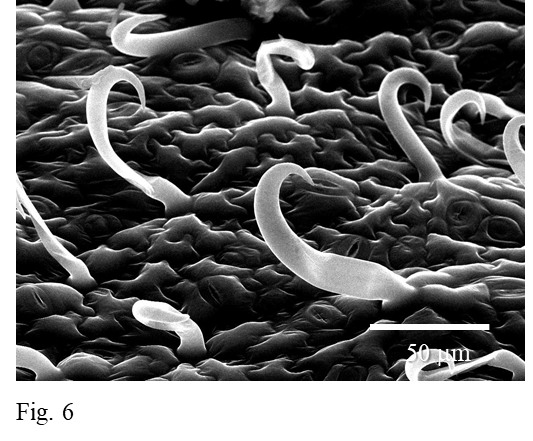
**Fig. S1. Hook-shaped trichomes on the abaxial surface of a true leaf.**

Hook-shaped trichomes are densely distributed and oriented towards the leaf surface. Scale bar: 50 µm.


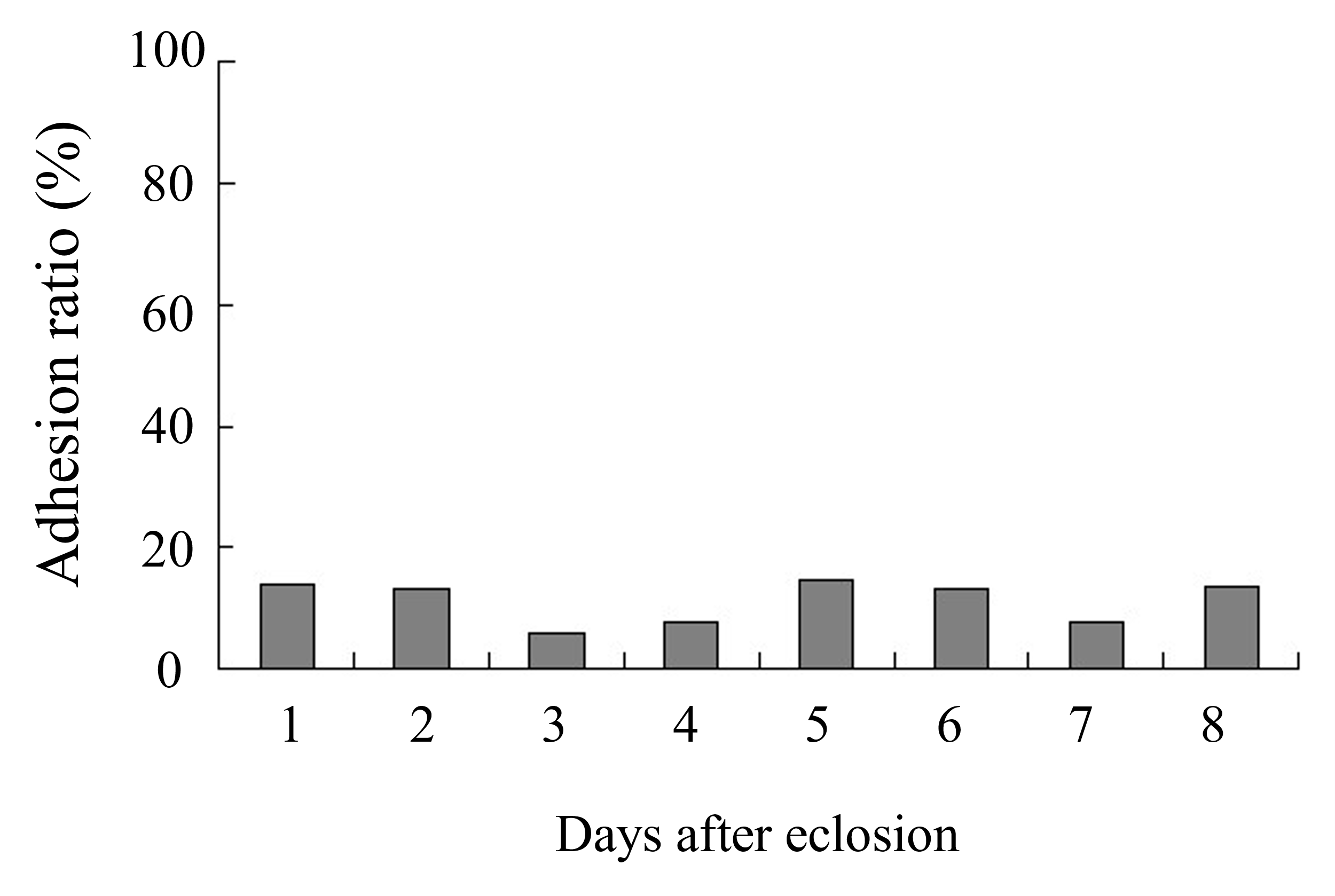
**Fig. S2. Effects of age on trichome-mediated attachment in *L. trifolii*.**

Attachment (adhesion) ratios of adults measured on each day from 1 to 8 days post-eclosion. No significant trend in attachment was observed across ages (χ^2^ test; P = 0.7303). The average attachment rate remained approximately 15% throughout.


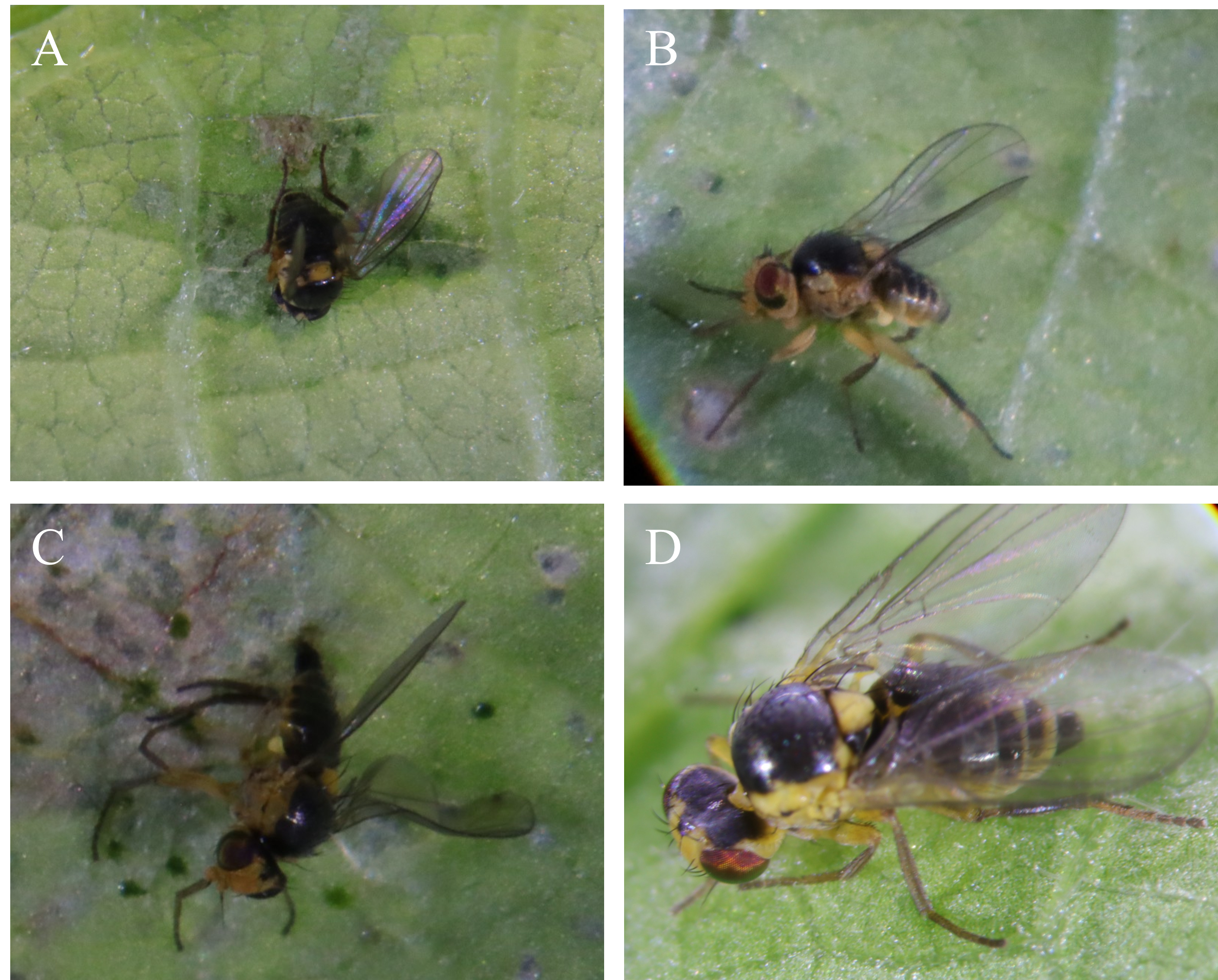
**Fig. S3. *Liriomyza trifolii* individuals trapped by leaf trichomes.**

(A) Individual with legs and mouthparts entrapped and deceased. (B) Individual with the lateral side of a leg entrapped. (C) Deceased individual with legs entrapped and desiccated. (D) Living individual immediately after leg entrapment.


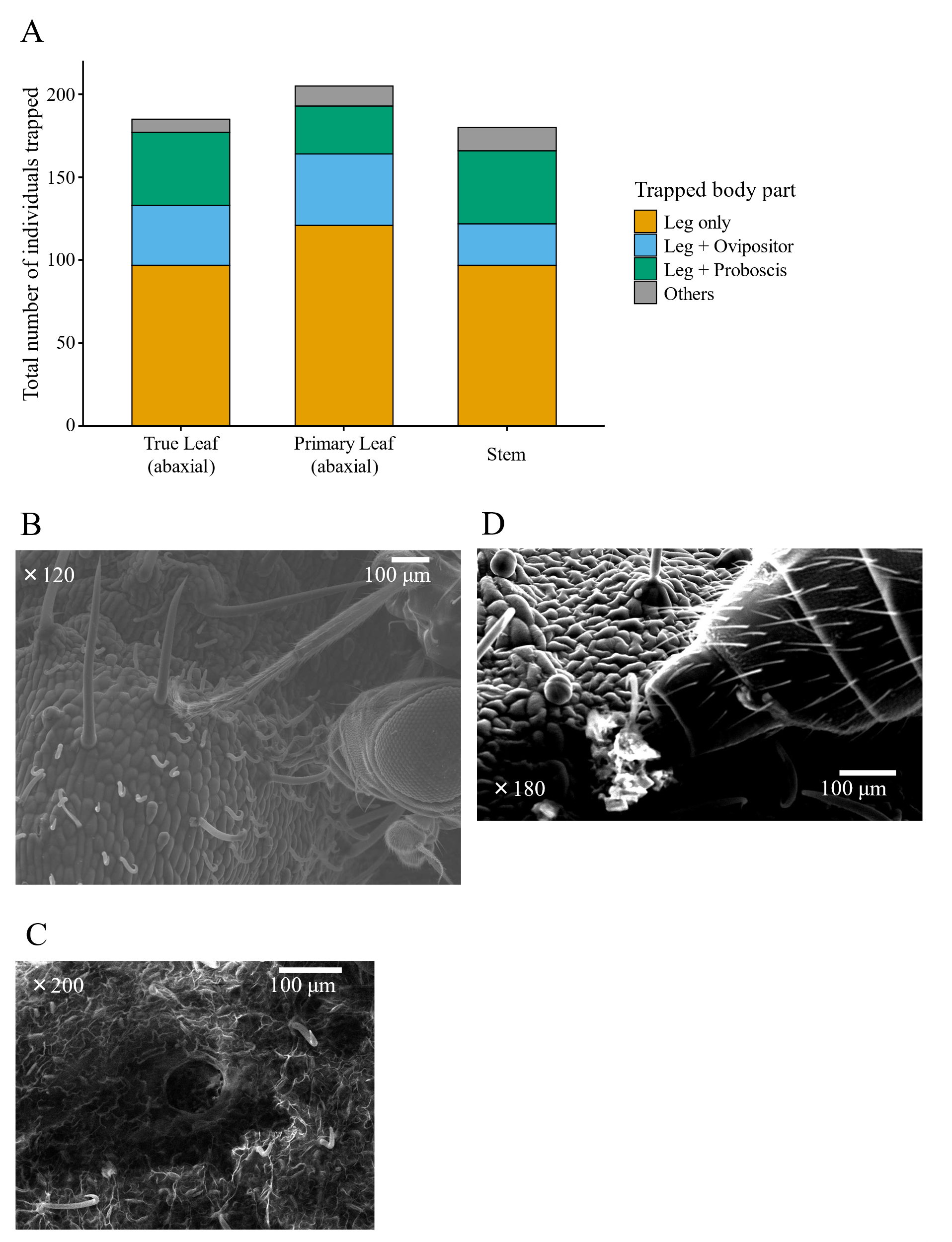
**Fig. S4. Detailed analysis of trichome entrapment modes in leafminers.** (A) Breakdown of trapped body parts of *L. trifolii* on the abaxial surfaces of true leaves, primary leaves, and stems. Stacked bars represent the number of individuals trapped by legs only (orange), legs and ovipositor (blue), legs and proboscis (green), or other parts (gray). Data correspond to the species-specific attachment analysis shown in Figure 5. (B) Representative stereomicroscope image of an *L. trifolii* adult with its proboscis (mouthpart) entrapped by a hook-shaped trichome. The white scale bar represents 100 µm. (C) Close-up of an *L. trifolii* female with her ovipositor entrapped by trichomes. The white scale bar represents 100 µm. (D) Oviposition punctures created by *Chromatomyia horticola* on the abaxial surface of a kidney bean leaf. The white scale bar represents 100 µm.

**Video S1.**

A live *L. trifolii* individual attempting to escape after its leg is caught by a hook-shaped trichome on the abaxial surface of a true leaf.
